# Central Systolic Hypertension in Patients with Well-Controlled Hypertension

**DOI:** 10.1155/2017/8158974

**Published:** 2017-01-03

**Authors:** Jozef Bulas, Mária Potočárová, Ján Murín, Katarína Kozlíková, Ján Luha, Martin Čaprnda

**Affiliations:** ^1^1st Department of Internal Medicine, Comenius University, Faculty of Medicine, Mickiewiczova 13, 813 69 Bratislava, Slovakia; ^2^Institute of Medical Physics, Biophysics, Informatics and Telemedicine, Comenius University, Faculty of Medicine, Sasinkova 2, 813 72 Bratislava, Slovakia; ^3^Institute of Medical Biology, Genetics and Clinical Genetics, Comenius University, Faculty of Medicine, Sasinkova 4, 813 72 Bratislava, Slovakia

## Abstract

*Background*. Central systolic blood pressure (CSBP) has prognostic significance and simplified devices for its estimation have been introduced recently. The aim of this study was to assess the achievement of the target CSBP in treated hypertensive patients.* Subjects and Methods*. One hundred patients with well-controlled hypertension were analysed. For CSBP estimation, we used the Arteriograph (TensioMed Ltd.), which uses one cuff for all measurements, the “single-point measurement” approach.* Results*. We found that 62% of patients had CSBP ≥ 130 mmHg, the suggested cut-off value for hypertension. When sex-specific classification was employed (CSBP ≥ 137 mmHg for female and CSBP ≥ 133 mmHg for male), only 13% of patients (mainly women) remained in the hypertensive range. We also found that 55% of patients had a CSBP higher than brachial pressure. Multiple analyses showed that CSBP was significantly associated with sex, height, and return time.* Conclusions*. A high proportion of treated hypertensive patients had CSBP levels that exceeded their brachial BP. CSBP positively correlated with lower height and shorter return time of the reflected pressure wave and was significantly higher in females compared to males. These findings suggest that, for CSBP classification, it is important to take height and sex-specific differences into account.

## 1. Introduction

Arterial hypertension is the most important modifiable cardiovascular risk factor. Blood pressure (BP) measurement has been extensively used for more than a century, and brachial cuff BP has been used for the diagnosis and treatment of hypertension. Consequently, guidelines and classifications are based on this approach [1, 2].

Systolic BP is a result of the interaction between ejected stroke volume and the dampening function of large arteries and propagative and reflected pressure waves. Both pulse pressure and systolic pressure increase due to pulse pressure amplification as pressure waves propagate from the heart to the periphery, and from where the pressure waves reflect back to the heart [3, 4].

Early return of reflected pressure waves (in the case of stiffer arteries) may increase central systolic pressure in the aortic root during late systole and may increase the workload of the left ventricle [5]. Standard BP measurement using the brachial artery does not allow for the estimation of central BP and the ascertainment of the real work of the left ventricle [6, 7].

Central systolic blood pressure (CSBP) is considered an important haemodynamic parameter, and several large studies indicate that it is an important prognostic risk factor [8]. Cardiovascular events decrease with lower CSBP levels, and this finding explains the differences between patient subgroups with similar brachial BP but different CSBP; lower CSBP levels resulted in more favourable clinical outcomes [9–11].

The standard for noninvasive central BP estimation is generally accepted to be applanation tonometry, in which pulse waves on the radial or carotid arteries are detected by a sensitive probe. The pulse wave curve obtained requires calibration, and this is achieved by entering the BP values from brachial BP measurements and is corrected by the transfer factor. The peripheral pressure waveform is then intertwined with the wave in the ascending aorta [3, 12]. Such an examination is methodologically challenging (needs specialized equipment and training) and time-consuming and requires a high degree of expertise [12, 13].

Newer and simpler methods rely on cuff-based waveform analyses, in which pressure waves and BP measurements are taken by a single BP cuff on the arm. This technique, called single-point measurement, is operator-independent and such devices have shown some promise for routine clinical use [14]. The obtained pressure wave curve has a typical pattern that facilitates the analysis of BP changes and the evaluation of heart cycle timing.

For the research and clinical implication of vascular stiffness measurement, it is important to standardise the methodology of examination of arterial stiffness. Methodological aspects of stiffness measurements, wave reflections, central pulse pressure, and aortic pressure estimation are discussed in the Recommendation of the American Heart Association (AHA) 2015 [14]. Papers concerning “reference values” of pulse wave velocity (PWV, a marker of arterial stiffness) and of CSBP have been published recently [15–17]. Although the routine clinical use of CSBP measurement is under debate, growing evidence of the prognostic significance of such an approach supports its implementation in practice [18–20]. The continued improvement of the accuracy and reliability and the standardisation of methodology suggest that CSBP measurement will become more common in daily practice [7, 18].

In this study, we aimed to identify the potential benefits of CSBP measurement in the management of patients with hypertension in clinical practice. Thus, we analysed a group of patients who visited our outpatient department that specializes in providing care for patients with high BP. We also assessed how successful we were in achieving the recommended CSBP level according to data in the current literature and to compare CSBP readings to brachial BP in treated hypertensive patients.

## 2. Materials and Methods

For this analysis, we retrospectively selected data from 100 patients with well-controlled hypertension (brachial blood pressure below 140/90 mmHg) from a group of 199 consecutive patients referred to our outpatient department for further investigation or for a follow-up examination from May 2008 to December 2015. The patients were originally involved in a research project studying central haemodynamics and preclinical cardiovascular disease, which was approved by Ethical committee of University Hospital Commenius University Bratislava. The patients signed an informed consent form after we explained to them the nature and aim of this type of noninvasive BP measurement, which was added to our usual set of examinations devoted to the search for preclinical cardiovascular diseases in patients diagnosed with hypertension. The patients selected for this analysis had undergone a basic clinical examination and a recent echocardiographic examination. They had a blood pressure (measured by arteriography) below 140/90 mmHg and their current medication list was known. The exclusion criteria were as follows: atrial fibrillation, systolic or diastolic BP above the target values, left ventricular systolic dysfunction, heart failure, unstable angina or clinically significant valve disease, advanced renal failure [glomerular filtration rate (GFR) < 60 ml/(min·m^2^)], and/or significantly increased liver function test values (liver function test values greater than 3 times the upper normal limit).

Each patient underwent standard clinical evaluation, measurement of anthropometric parameters, laboratory tests including lipid profile, and echocardiography with the measurement of basic parameters such as left ventricular wall thickness and dimensions, systolic function, calculated left ventricular mass (LVM) according to Devereux et al. [21], LVM indexed to the body surface area (LVM_i_), relative wall thickness, and diastolic function. The threshold for left ventricular hypertrophy (LVH) was considered as the left ventricular mass index, LVM_i_ ≥ 95 g/m^2^ for women and LVM_i_ ≥ 115 g/m^2^ for men [1]. Specifically, patients with well-controlled BP, below 140/90 mmHg, at the time of examination (recommended goal arm BP) were included in the analysis.

The invasively and noninvasively validated instrument, Arteriograph Tensiomed Ltd. (H-1103 Budapest, Hungary), was used to estimate the central BP [20, 22–25]. It works based on the oscillometric principle, using only one cuff for both BP measurement and waveform detection for pressure wave analysis (one measurement site, single-point analysis) [14]. The cuff for BP and central haemodynamic evaluation was tightly fastened on the dominant arm above the elbow as recommended in the user's manual [26]. The pressure wave is self-calibrated using the brachial pressure, which is obtained during the same measurement cycle [26]. One measurement cycle lasts 2 to 3 minutes. Patients were examined after 5 to 10 minutes of rest in the supine position. After placing the cuff in the proper position on the arm, the actual measurement is automated and operator-independent, and results of measurements depend solely on the measuring device. Because of the inherent variability of blood pressure, [13] some differences were expected between repeated measurements. A two-way ANOVA on these repeated measurements revealed significant differences between the first and third measurements, but not between the second and third of the three repeated measurements in one session. For evaluation of the reliability of measurements, we measured the concordance of the direction of the differences between the CSBP and sBP-brachial (higher or lower CSBP). From 41 tested measurements, only five patients showed a divergence in the direction of differences. In our cohort, 88% of all patients showed concordance in the direction of the differences between CSBP and sBP-brachial (all three values of CSBP measured in one patient were either higher or lower than values of sBP-brachial). The opposite direction of differences was present mainly in patients with small (near to zero) CSBP to sBP-brachial differences.

The Arteriograph Tensiomed provides several parameters of peripheral and central haemodynamics; the most important are brachial systolic and diastolic pressure, pulse pressure, heart rate, CSBP, brachial and aortic augmentation indexes, duration of left ventricular ejection, diastolic reflection area, and systolic and diastolic area indexes (see Abbreviations).

We classified CSBP according to the values published by Cheng et al. in 2013 [16]. The threshold for optimal central BP was less than 110/80 mmHg, for prehypertension it was 110–129/80–89 mmHg, and for hypertension it was greater than or equal to 130/90 mmHg. For the cut-off value for normal PWV, we used a speed of 10 m/s as recommended in the 2013 ESC/ESH guidelines [1].

For data processing, we used the mean values from two to three subsequent measurements or the measurement with the lowest standard deviation of PWVao value provided by the Arteriograph. Obtained data were submitted for statistical evaluation (IBM SPSS Statistics 23), testing first for normality of distribution using the Shapiro-Wilk test. For comparisons of nonnormally distributed data, we used the Mann–Whitney *U*-test, *χ*^2^ test in contingency tables, and Fisher's exact test. For comparisons of selected parameters, we used the *t*-test. For testing of relationships between selected parameters we used Pearson and Spearman's correlation. Multivariate stepwise regression analysis was used for verifying significant correlation of CSBP with important parameters. A *p* value < 0.05 was selected as the threshold for significant differences.

## 3. Results

### 3.1. Patient's Characteristics

We evaluated 100 patients (38 males and 62 females) with an average age of 64.0 ± 10.7 years. Women were significantly older than men were, were shorter, had a lower body mass index (BMI), and had nonsignificantly higher systolic brachial BP than men (Table 1). Basic laboratory values can be found in Table 2. LVH according to the sex-specific scale for LVM_i_ was significantly more prevalent in females (Table 3).

Risk factors for cardiovascular diseases, such as dyslipidemia (in 89% of patients), impaired glucose tolerance (21%), type 2 diabetes mellitus (T2DM, 24%), and obesity (31%), were relatively common (Table 4). Frequently occurring comorbidities were stable coronary artery disease (43%), mild renal dysfunction [GFR, 60–90 mL/(min·m^2^)] in 31%, history of transient ischaemic attack or stroke (10%), and PAD (4%) (Table 4). All patients were treated mostly by a combination of antihypertensive drugs; angiotensin converting enzyme inhibitors (ACEI), calcium channel blockers (CCB), hydrochlorothiazide-type diuretics (D), and beta-adrenergic blocking drugs (BB) were the most frequently used antihypertensives (Table 5).

### 3.2. Central Systolic BP and Aortic Stiffness

All patients in the study group were well-treated and achieved the recommended goal for brachial BP (<140/90 mmHg), as measured by the Arteriograph in each measurement cycle. Mean systolic brachial BP in the whole group was 123.6 ± 10.1 mmHg; the values for women were higher than those for men, but the differences were not significant. CSBP was significantly higher in females compared to males (Table 6).

The mean value of the estimated CSBP in the aorta was 123.2 ± 12.1 mmHg, but almost two-thirds of patients (62%) had CSBP ≥ 130 mmHg consistent with central hypertension according to the proposed classification for central BP published in JACC 2013 [16]. The mean CSBP in the central hypertensive subgroup was 130.6 ± 7.5 mmHg while that in the central nonhypertensive subgroup was CSBP 111.0 ± 7.1 mmHg (Table 7, part A).

When we used another, sex-specific (but more complex) classification suggested by a “Working Group for Central Systolic Blood Pressure” published in EHJ 2014 [17], only 13% of patients remained in the hypertensive level. Dividing patients into subgroups according to these cut-off values for hypertension (men: CSBP ≥ 133 mmHg; women: CSBP ≥ 137) showed that patients with higher CSBP had lower height and weight and were nonsignificantly older; these characteristics were more prevalent in women (Table 7, part B). PWVao was nonsignificantly faster (10.2 m/s versus 9.4 m/s) and the aortic and brachial augmentation indexes were significantly higher among hypertensive patients. The brachial systolic BP-to-central systolic BP ratio was significantly lower in the hypertensive patients (Table 7, part B). Using simple analysis we found a significant correlation of CSBP with lower height (*r* = −0.299, *p* = 0.003), shorter RT (*r* = −0.324, *p* = 0.001), and higher PVVao (*r* = 0.223, *p* = 0.026). Multiple analyses (model with age, height, weight, RT, PWVao, and sex) showed that CSBP was significantly dependent on sex and return time. After removing sex from the model, the height became a significant parameter. After dividing patients according to sex, the PWVao was significant in both sexes, the RT was significant in women, and age was significant in males (Table 8).

When we compared the central and brachial systolic BPs, we discovered that more than half of our patients (55%) had a higher central systolic BP than brachial BP; mean central systolic BP values in these subgroups were 129 ± 10.3 mmHg for the subgroup with higher central systolic than brachial BP versus 116 ± 10.1 mmHg for the subgroup with lower central systolic than brachial BP (Table 7, part C). The PWVao in the subgroup with higher central than brachial systolic BP was faster (9.8 ± 1.8 m/s versus 9.2 ± 1.6 m/s in the subgroup with higher central systolic than brachial BP, *p* value = 0.064); brachial augmentation index (17.3 ± 15.4% versus −21.1 ± 17.1%, *p* < 0.001) and aortic augmentation index (40.0 ± 5.7% versus 23.7 ± 7.3%, *p* < 0.001) were higher and return time was shorter (106.8 ± 18.7 ms versus 121.8 ± 22.2 ms, *p* < 0.001) in the group with higher central systolic than brachial BP compared to those in the group with lower central systolic than brachial BP, respectively. Of the 55 persons in this subgroup, 44 were female (80%), and they were older, of shorter stature, and with lower BMI; the differences were significant. An overview of arterial stiffness parameters and related variables are provided in Table 7, part C.

Because of the large proportion of women in the group of patients with high CSBP, we statistically evaluated the group based on the sex distribution. The PWVao was nonsignificantly faster in women. However, they had a significantly higher CSBP (126 mmHg versus 118 mmHg), shorter return time (RT), and higher augmentation indexes than men (Table 6).

Antihypertensive therapy was based on a combination of antihypertensive drugs. The mean count of antihypertensive agents per person was 2.6 ± 1.2 (Table 5). We did not find any significant influence of specific drugs on CSBP, the class of drugs, the combinations of drugs, or the number of drugs used. In the laboratory parameters, no important correlations were noted except for a significantly lower GFR in the group with higher CSBP than brachial BP (Table 7, Part C). LVH was significantly more frequent in the group with CSBP ≥ 130 mmHg (Table 7, Part A).

## 4. Discussion

In this study, we evaluated CSBP in treated hypertensive patients. Unexpectedly high was the proportion of patients who had CSBP levels higher than brachial SBP. These findings are different from most published data, which show the brachial systolic BP higher than CSBP because of peripheral pressure wave amplification [13, 27]. For the estimation of central BP, we used the Arteriograph Tensiomed^Ltd^. The Arteriograph was validated based on the finding of a strong correlation between invasively measured and noninvasively estimated CSBP [20]. Rossen et al. [22] found a mean difference of 4.4 mmHg, where the Arteriograph underestimated the central BP against invasively measured values. Several studies that compared the Arteriograph with other noninvasive methods for arterial stiffness estimation have been published, mainly compared to tonometric (Sphygmocor) or piezo-electronic devices (Complior). Good agreement was also achieved for PWVao estimation. The correlations of PWVao assessed with the Arteriograph and with the Sphygmocor (*r* = 0.67, *p* < 0.001) and the Complior (*r* = 0.69, *p* < 0.001) were considerable, but the techniques are not interchangeable and, therefore, for follow-up measurements, it is necessary to use the same device [23–26, 28]. The variability and reproducibility for PWV were best for the Arteriograph followed by Complior and Sphygmocor [25].

In this study, each patient achieved the target brachial BP (<140/90 mmHg). If we accept the suggested cut-off value of greater or equal to 130 mmHg for the definition of central systolic hypertension [16], we can also analogically assume this value as the target for treated CSBP. CSBP below this value (<130 mmHg) was not attained by 62% of our patients, while the target for brachial BP was achieved by all patients. This finding resembles the results published by McEniery (Anglo-Cardiff Collaborative trial II) who described that 78% of men and 72% of women with high-normal brachial BP (systolic BP < 140 mmHg) had similar aortic systolic BP as patients with brachial systolic pressures in stage I hypertension, indicating that they had overlapping values of CSBP [29].

It is known that central haemodynamics depends on aortic stiffness and reflections of pressure waves from the peripheral arterial tree and is influenced by risk factors and cardiovascular diseases [30].

We analysed the interdependencies between PWVao (as an indicator of aortic stiffness) and CSBP. According to all criteria used for central hypertension, hypertensive patients, in comparison to normotensives, had only nonsignificantly higher PWV; however, the return time of the reflected wave into the aortic root was significantly shorter in hypertensive patients. This discordance between the velocity of the aortic pulse wave (which was similar in both groups) and the shorter return time of the reflected wave may be explained by a shorter distance (thus, with a shorter return time) of the reflected pulse wave to reach the central aorta [30], as the patients in the subgroup with central systolic hypertension according to sex-specific criteria and in the group of higher CSBP comparing to brachial SBP were significantly shorter (Table 7, parts B and C). This relationship was only borderline in the subgroup with CSBP higher than 130 mmHg (Table 7, part A). These findings are in agreement with the literature, which shows lower cardiovascular mortality in taller individuals and higher cardiovascular risk in shorter persons [31].

The increase of augmentation pressure, pulse pressure, and late systolic pressure due to shorter return time may increase left ventricular load and can predict heart failure [32]. In agreement with this explanation, the ejection duration from the left ventricle has been significantly prolonged in the patients with central systolic BP higher than brachial systolic BP (330.9 ms versus 315.7 ms) (Table 7, part C). We can assume that prolongation of ED concurrently widens the pulse pressure, which leads to ventricular hypertrophy [33].

When we applied the sex-specific scale for CSBP classification (≥133 mmHg for males, ≥137 mmHg for females) presented in EHJ in 2014 [17], which is more specific in comparison to the simpler classification published in JACC in 2013 (cut-off value for hypertension ≥ 130 mmHg for both sexes) [16], the percentage of patients in the hypertensive range dropped from the initial 62% to only 13%. The reclassification to normotension level involved more men than women.

Because females were more frequently among the centrally hypertensive, we also evaluated our patients from a sex-specific aspect. Women had significantly higher CSBP (126 mmHg versus 118 mmHg), lower height (163 cm versus 178 cm), and higher age (66 years versus 61 years) than men (Tables 1 and 6). These results are in agreement with published data that show that central BP increases more with age, compared to peripheral systolic BP, and that this increase is steeper in women than in men [30]. The accuracy of CSBP estimation depends strongly on blood pressure values used for pressure wave calibration. Cheng and coworkers found substantial improvement of accuracy of noninvasively (applanation tonometry) obtained CSBP when invasively measured BP was used for calibration of the peripheral waveform in comparison to results when cuff BP was used for calibration; cuff BP-based calibration underestimated the CSBP by −8.2 mmHg [34]. Costello et al. [35] have tested a new, brachial cuff-based (suprasystolic) technique for estimation of CSBP (a method similar to the Arteriograph) and found an underestimation of central BP by −7 mmHg in comparison to invasive measurements.

To avoid problems with BP calibration, Benetos et al. [36] in their study published in 2010 suggested the use of the ratio of the carotid/brachial pulse pressure to predict cardiovascular mortality, and they found that this ratio is less dependent on BP calibration and may be directly applicable in large population studies. In our study, we found that the ratio of central to brachial systolic BP in our patients had similar statistical significance as the BP differences (Table 7).

Among the basic echocardiographic parameters, we found that LVH significantly occurred more frequently in women than in men (53% versus 29%) (Table 3). LVH also occurred more frequently in patients with CSBP ≥ 130 mmHg than in patients with CSBP < 130 mmHg (55% versus 26%) (Table 7, Part A).

In a large proportion of our patients (55%), CSBP was higher than brachial systolic BP. In the literature, the prevalent opinion is that CSBP is lower compared to brachial BP because of peripheral BP amplification by wave reflections [13, 27, 33]. Safar et al. [37] found a peripheral amplification, which ranged approximately from 7 to 11 mmHg, and amplification was increased with higher brachial BP. Augmentation, which is the increase of central pressure due to the summation of the reflected pressure wave with the primary pressure wave, usually does not exceed the peripheral systolic pressure. The increase of central pressure due to considerable augmentation is usually referred to as the decrease of amplification [37].

Some results have been published suggesting the augmentation of CSBP above the brachial BP values. Munir et al. [38] emphasised the direct relationship between amplification and augmentation based on their findings that the late systolic shoulder of peripheral pressure waveform closely approximates central systolic pressure and that the peripheral augmentation index was related to central systolic pressure. They found a close agreement between the late systolic shoulder of the peripheral pulse and invasively measured values of central systolic pressure, with a mean difference of 0.5 ± 5.2 mmHg, indicating that in some proportion of patients the invasively measured CSBP exceeds peripheral systolic BP even though the mean value of noninvasively measured brachial SBP was higher than CSBP. These results are compatible with the formation of either the type D pressure wave contour in the ascending aorta where the reflected wave arrives early in systole and merges with the incidental pressure wave or the type A configuration where peak systolic pressure occurs in late systole, with augmentation index greater than 12% [39].

This situation is frequently present in clinical states, which increase the degree of aortic stiffness, such as hypertension, diabetes mellitus, or renal failure or due to natural age-dependent changes [40]. In recent studies, CSBP appears to be an important independent risk factor for cardiovascular disease and mortality and, therefore, antihypertensive treatment should be focused not only on the decrease of brachial BP but also on the lowering of CSBP [41–45].

Antihypertensive drugs decrease both brachial BP and CSBP but they differ in the magnitude of decreasing the CSBP, as was shown in a meta-analysis by Manisty and Hughes [27]. They analysed results of several studies and found significant heterogeneity between drug classes used either as monotherapy or in combinations. Combinations of ARBs with CCBs, ARBs with Ds, and ACEIs with CCBs reduced CSBP and brachial SBP to similar extents. Beta-blockers, diuretics, and combinations containing beta-blockers tended to reduce central to brachial amplification, implying that the decrease of brachial SBP is associated with lesser reduction in CSBP. This could explain the differences in clinical outcomes of trials comparing beta-blocker and diuretic-based therapy with other regimens [10, 27]. Beta-blockers with vasodilating properties (such as nebivolol and carvedilol) are reported to have more beneficial effects [14]. In the CAFE study [10] the combination of amlodipine with ACEI achieved better outcomes in comparison to atenobene with diuretic. This difference was explained by a greater decrease of CSBP in the amlodipine + ACEI group (this difference was 4.3 mmHg). In the EXPLOR Study, the combination of amlodipine and valsartan lowered CSBP to a larger extent than amlodipine and atenolol in combination [46]. Among our patients who were mostly on combination therapy, we did not find any significant influence of drugs or their combination on CSBP. Evaluation of the effects of drug therapy on CSBP would require larger studies.

## 5. Limitations

This study was a retrospective analysis of treated hypertensive patients who had achieved the recommended target arm blood pressure values. One limitation of the study was that patients were treated with different antihypertensive drugs. Most frequently, ACE inhibitors or ARBs were used in combination with calcium channel blockers and/or diuretics; beta-blockers were also frequently used. The average number of antihypertensive agents per person was 2.6 ± 1.2. In this study, central systolic hypertension was more frequently detected in women. The women in our analysis were older and shorter than the men; these factors likely contributed to higher CSBP. Due to the combination therapy and relatively small number of patients in our study, we did not find the influence of specific drugs on CSBP. However, the purpose of this study was more focused on comparing central and brachial systolic BP in well-controlled hypertension.

## 6. Conclusions

We found important differences between brachial and CSBPs in our group of patients with well-controlled hypertension, defined as an arm BP below 140/90 mmHg. A significantly large proportion of these patients have high CSBP. While the brachial pressures in the whole group were within the target range, 62% of patients had estimated CSBPs above the proposed cut-off value of 130 mmHg for central systolic hypertension. Using the sex-specific European proposal for CSBP classification, only 13% of patients (mainly women) remained in the hypertensive range. When we used another approach and compared the central systolic and brachial systolic BPs for each individual, we found that the estimated CSBP was higher than the brachial systolic BP in 55% of patients.

Factors that significantly contributed to higher CSBP included shorter height, older age, and female sex. Generally, women are of shorter stature, which significantly influences the timing of pressure wave propagation and reflection. In CSBP classification, factors such as sex, age, and height should be taken into account. However, this approach requires more complex classification scales. Such a different approach can be seen in recently published papers in the Journal of the American College of Cardiology 2013 [16] and European Heart Journal 2014 [17]. CSBP is an important parameter characterising central haemodynamics and is expected to become the therapeutic target in patients with arterial hypertension. The proportion of our patients in whom CSBP exceeded the brachial SBP was unexpectedly high. This phenomenon has not been clearly described in the literature and its clinical significance deserves attention specifically in evaluation of hypertension treatment.

## Figures and Tables

**Table 1:** Basic clinical characteristics of the subjects.

	All	Males	Females	*p* value
Number of patients (*n*)	100	38	62	
Age (years)	64.0 ± 10.7	60.6 ± 11.0	66.1 ± 10.0	*p* = 0.018
Height (cm)	169.3 ± 9.2	178.4 ± 5.0	163.6 ± 6.1	*p* < 0.001
Weight (kg)	80.2 ± 15.4	93.3 ± 13.0	72.2 ± 10.7	*p* < 0.001
BMI (kg/m^2^)	27.9 ± 3.9	29.3 ± 3.8	27.0 ± 3.7	*p* = 0.010
BSA (m^2^)	1.9 ± 0.2	2.1 ± 0.1	1.8 ± 0.1	*p* < 0.001
sBP-brachial (mmHg)	123.6 ± 10.1	122.5 ± 10.7	124.2 ± 9.8	*p* = 0.414
dBP-brachial (mmHg)	74.9 ± 6.9	75.3 ± 6.9	74.7 ± 7.0	*p* = 0.803
PP-brachial (mmHg)	48.8 ± 7.3	47.2 ± 6.7	49.8 ± 7.5	*p* = 0.137
Heart rate (b/min)	62.6 ± 8.5	62.6 ± 9.0	62.6 ± 8.3	*p* = 0.899

Data are given as mean ± standard deviation.

sBP: systolic blood pressure, dBP: diastolic blood pressure, and PP: pulse pressure.

BMI: body mass index. BSA: body surface area.

**Table 2:** Basic laboratory values.

	Mean ± SD
S_glucose (mmol/l)	5.7 ± 1.2
S_creatinine (*μ*mol/l)	77.1 ± 17.2
S_urea (mmol/l)	5.9 ± 1.7
S_uric acid (*μ*mol/l)	312.3 ± 78.9
GFR (ml/(min·m^2^))	81.3 ± 23.3
S_total cholesterol (mmol/l)	4.9 ± 1.0
S_LDL cholesterol (mmol/l)	3.3 ± 2.1
S_HDL cholesterol (mmol/l)	1.4 ± 0.4
S-TAG cholesterol (mmol/l)	1.5 ± 1.1

S: serum, LDL: low-density lipoprotein, HDL: high-density lipoprotein, TAG: triglyceride, GFR: glomerular filtration rate.

**Table 3:** Basic echocardiographic parameters.

Parameter	Males	Females	*p* value
Left ventricular dimension (cm)	5.1 ± 0.4	4.7 ± 0.4	n/a
LVM (g)	230.5 ± 45.6	172.8 ± 45.6	n/a
LVM_i_ (g/m^2^)	108.7 ± 18.1	96.8 ± 22.8	n/a
Relative wall thickness. RWT	0.41 ± 0.05	0.39 ± 0.05	*p* = 0.205
Left ventricular EF (%)	58.6 ± 4.4	60.3 ± 4.1	*p* = 0.024
Left ventricular hypertrophy, LVH	11 patients (29%)	33 patients (53%)	*p* = 0.023

Data are given as mean ± standard deviation.

LVM: left ventricular mass, LVM_i_: left ventricular mass per m^2^ of body surface area (g/m^2^), RWT: relative wall thickness-ratio of wall thickness to internal dimension of left ventricle (RWT = IVS + LVPW/LVID), LVH: left ventricular hypertrophy-LVM_i_ ≥ 95 g/m^2^ in women or LVM_i_ ≥ 115 g/m^2^ in men, and n/a: not applicable due to sex differences of reference values.

**Table 4:** Cardiovascular risk factors and comorbidities.

	Incidence
*Cardiovascular risk factors*	
Dyslipidemia	89%
Obesity (BMI > 30 kg/m^2^)	31%
Renal dysfunction [GF 60–90 ml/(min·m^2^)]	31%
Impaired glucose tolerance (IGT)	21%
Diabetes mellitus type 2 (T2DM)	24%
Combination of IGT + T2DM	45%
Smoking	5%
*Comorbidities*	
Coronary artery disease	43%
Transient ischaemic attack/stroke	10%
Retinopathy	11%
Chronic heart failure	8%
Peripheral obliterative artery disease (PAD)	4%

BMI: body mass index.

**Table 5:** Antihypertensive and hypolipidemic treatment.

Number of drugs per patient (*n*, mean ± SD)	2.6 ± 1.2
Percentage distribution of antihypertensive drugs used	

ACE inhibitors, ACEI (%)	55
Calcium channel blockers (dihydropyridines), CCB (%)	55
Diuretics, D (%)	54
Beta-blockers, BB (%)	52
Angiotensin II receptor blockers (ARBs) (%)	21
Centrally acting antihypertensives (%)	12
Calcium channel blocker nondihydropyridine, verapamil (%)	9
Alpha-adrenoreceptor blocking drugs, AB (%)	6
Urapidil (%)	6
Statin therapy (%)	53
Fibrate therapy (%)	11

**Table 6:** Differences in arterial stiffness and related variables between men and women.

	Whole group	Males	Females	*p* value
Number of patients (*n*)	100	38	62	
PWVao (m/s)	9.6 ± 1.8	9.3 ± 1.9	9.7 ± 1.7	*p* = 0.137
RT (ms)	113.5 ± 21.6	124.1 ± 24.4	107.0 ± 16.8	*p* < 0.001
Aix brachial (%)	0.04 ± 25.1	−16.2 ± 24.1	10.0 ± 20.0	*p* < 0.001
Aix aortic (%)	32.7 ± 10.3	25.9 ± 10.5	36.8 ± 7.8	*p* < 0.001
Central systolic BP (mmHg)	123.2 ± 12.1	118.3 ± 10.8	126.1 ± 11.9	*p* < 0.001
Brachial systolic BP (mmHg)	123.6 ± 10.1	122.5 ± 10.7	124.2 ± 9.8	*p* = 0.414
Brachial/central SBP ratio	1.00 ± 0.05	1.04 ± 0.05	0.99 ± 0.04	*p* < 0.001
CSBP > brachial SBP (*n*, %)	55 (55%)	11 (29%)	44 (71%)	*p* < 0.001
Δc-bSBP (mmHg)	−0.41 ± 5.9	−4.1 ± 6.3	+1.87 ± 4.4	*p* < 0.001
Pulse pressure (mmHg)	48.8 ± 7.3	47.2 ± 6.7	49.8 ± 7.3	*p* = 0.137
Heart rate (b/min)	62.6 ± 8.5	62.6 ± 9.0	62.6 ± 8.3	*p* = 0.899
ED (ms)	324.1 ± 25.4	320.7 ± 24.5	326.1 ± 25.9	*p* = 0.290
DRA	41.2 ± 12	46.4 ± 13.6	38.0 ± 9.8	*p* < 0.001

PWVao: pulse wave velocity in the aorta; Aix brachial: brachial augmentation index; Aix aortic: aortic augmentation index; ED: duration of ejection from left ventricle; RT: return time; CSBP: central systolic blood pressure = systolic blood pressure in the aorta; Δc-bSBP: difference between central and brachial systolic blood pressure. DRA: diastolic reflection area.

**Table 7:** Aortic stiffness parameters. Division of patients into subgroups according to blood pressure differences. (A) Higher or lower CSBP than the cut-off value of 130 mmHg for central systolic hypertension (equal for women and men). (B) Sex-specific classification: hypertension in women = CSBP ≥ 137 mmHg; hypertension in men = CSBP ≥ 133 mmHg. (C) Lower CSBP than brachial systolic blood pressure or higher CSBP than brachial blood systolic pressure.

	Whole group	(A)			(B)			(C)		
		Division according to cut-off value 130 mmHg			Division according to sex-specific criteria of CSBP			Division according to CSBP to brachial BP		
		Normotension	Hypertension	*p* value	Normotension	Hypertension	*p* value	Lower CSBP than brachial systolic BP	Higher CSBP than brachial systolic BP	*p* value
		CSBP < 130 mmHg	CSBP ≥ 130 mmHg		Male CSBP < 133	Male CSBP ≥ 133				
					Female CSBP < 137	Female CSBP ≥ 137				
Number of patients (*n*)	100	38	62		87	13		45	55	

Males/females (*n*)	38/62	19/19	19/43	*p* = 0.060	37/50	1/12	*p* = 0.016	27/18	11/44	*p* < 0.001
Age (years)	64.0 ± 10.7	64.2 ± 11.7	63.9 ± 10.1	*p* = 0.730	63 ± 11	69 ± 9	*p* = 0.082	61.2 ± 12.4	66.3 ± 8.5	*p* = 0.017
Height (cm)	169.3 ± 9.2	171.4 ± 10.0	167.9 ± 8.5	*p* = 0.103	170.3 ± 9.1	162.2 ± 7.1	*p* = 0.004	173.0 ± 9.3	166.2 ± 8.0	*p* < 0.001
Weight (kg)	80.2 ± 15.4	83.5 ± 16.2	78.2 ± 14.7	*p* = 0.081	81.9 ± 15.6	68.8 ± 8.1	*p* < 0.001	87.1 ± 17.4	74.6 ± 2.4	*p* < 0.001
BMI (kg/m^2^)	27.9 ± 3.9	28.3 ± 4.2	27.6 ± 3.9	*p* = 0.314	28.1 ± 3.9	26.2 ± 3.4	*p* = 0.106	28.9 ± 4.4	27.0 ± 3.3	*p* = 0.013
PWVao (m/s)	9.6 ± 1.8	9.3 ± 1.8	9.7 ± 1.7	*p* = 0.064	9.4 ± 1.7	10.2 ± 1.7	*p* = 0.099	9.2 ± 1.6	9.8 ± 1.8	*p* = 0.064
RT (ms)	113.5 ± 21.6	119.2 ± 22.3	110.1 ± 20.6	*p* = 0.018	115.4 ± 22.1	101.2 ± 13.3	*p* = 0.036	121.8 ± 22.2	106.8 ± 18.7	*p* < 0.001
Aix brachial (%)	0.04 ± 1.80	−14.8 ± 20.1	+9.2 ± 23.5	*p* < 0.001	−2.7 ± 25.5	+18.6 ± 9.9	*p* < 0.001	−21.1 ± 17.1	+17.3 ± 15.4	*p* < 0.001
Aix aortic (%)	32.7 ± 10.3	26.5 ± 8.9	36.4 ± 9.4	*p* < 0.001	31.3 ± 10.3	41.9 ± 4.2	*p* < 0.001	23.7 ± 7.3	40.0 ± 5.7	*p* < 0.001
CSBP	123.2 ± 12.1	111.0 ± 7.1	130.6 ± 7.5	*p* < 0.001	120.2 ± 10.0	142.7 ± 2.1	*p* < 0.001	116 ± 10.1	129 ± 10.3	*p* < 0.001
Brachial systolic BP	123.6 ± 10.1	114.9 ± 8.9	128.9 ± 6.5	*p* < 0.001	121.6 ± 9.3	136.7 ± 1.8	*p* < 0.001	121.9 ± 11.0	125.0 ± 9.2	*p* = 0.192
Δc-bSBP (mmHg)	−0.41 ± 5.9	−3.9 ± 4.2	+1.8 ± 5.3	*p* < 0.001	−1.4 ± 5.7	+5.9 ± 1.6	*p* < 0.001	−5.8 ± 4.2	+4.0 ± 2.3	*p* < 0.001
Brachial/central SBP ratio	1.01 ± 0.04	1.04 ± 0.05	0.99 ± 0.04	*p* < 0.001	1.01 ± 0.05	0.96 ± 0.01	*p* < 0.001	1.05 ± 0.04	0.97 ± 0.02	*p* < 0.001
Pulse pressure, PP (mmHg)	48.8 ± 7.3	46.7 ± 7.3	50.7 ± 6.6	*p* < 0.001	47.4 ± 6.4	58.1 ± 5.9	*p* < 0.001	45.1 ± 10.6	51.6 ± 9.7	*p* = 0.002
Heart rate (b/min)	62.6 ± 8.5	64.5 ± 8.3	61.4 ± 8.5	*p* = 0.032	62.8 ± 8.7	60.8 ± 0.6	*p* = 0.548	65.2 ± 8.5	60.4 ± 8.3	*p* = 0.005
ED (ms)	324.1 ± 25.4	318.3 ± 26.4	327.6 ± 24.2	*p* = 0.130	322.8 ± 25.6	332.2 ± 23.5	*p* = 0.232	315.7 ± 23.1	330.9 ± 25.2	*p* = 0.002
DRA	41.2 ± 12.0	43.5 ± 11.9	39.8 ± 11.0	*p* = 0.109	41.7 ± 11.9	37.7 ± 13.1	*p* = 0.469	45.8 ± 13	37.4 ± 9.8	*p* < 0.001
LVH (LVM_i_), *n* (%)	44 (44%)	10 (26%)	34 (55%)	*p* = 0.007	37 (43%)	7 (54%)	*p* = 0.553	19 (42%)	25 (46%)	*p* = 0.840
GFR [ml/(min/m^2^)]	81.3 ± 23.3	85.9 ± 27.1	78.4 ± 20.3	*p* = 0.184	82.9 ± 23.5	70.6 ± 20.2	*p* = 0.116	87.8 ± 25.9	76.2 ± 19.8	*p* = 0.015

PWVao: pulse wave velocity in the aorta; Aix brachial: brachial augmentation index; Aix aortic: aortic augmentation index; ED: duration of ejection from the left ventricle; RT: return time; CSBP: central systolic blood pressure = systolic blood pressure in the aorta; Δc-bSBP: difference between central and brachial systolic blood pressure. DRA: diastolic reflection area.

**Table 8:** Multivariate analysis. Influence of important parameters on CSBP.

Independent parameter	Models							
	All patients		All patients (model without sex)		Males		Females	
	Coeff. *B*	*p* value	Coeff. *B*	*p* value	Coeff. *B*	*p* value	Coeff. *B*	*p* value
Sex (M = 0, F = 1)	5.75	0.025	—	—	—	—	—	—

Age	−0.15	0.239	−0.165	0.186	−0.341	0.046	−0.008	0.960

Height	−0.073	0.750	−0.271	0.050	0.004	0.992	0.089	0.762

Weight (kg)	0.049	0.656	−0.069	0.522	−0.041	0.782	−0.095	0.559

RT (ms)	−0.119	0.039	−0.12	0.044	0.104	0.640	−0.507	0.007

PWVao (m/s)	−1.499	0.370	−1.594	0.337	2.764	0.008	−4.17	0.026

Coeff.: unstandardized regression coefficient.

## Abbreviations

sBP-brach: Brachial systolic blood pressure
dBP-brach: Brachial diastolic blood pressure
PP: Pulse pressure
MAP: Mean arterial pressure
HR: Heart rate
CSBP: Central systolic blood pressure (in the ascending aorta)
PPao: Pulse pressure in the ascending aorta
Aix aortic: Aortic augmentation index
Aix brachial: Brachial augmentation index ((*P*_2_ − *P*_1_)/PP)
PWVao: Aortic pulse wave velocity, the estimated velocity of the aortic pulse wave
RT: Return time, the time between the peak of the primary pressure wave (*P*_1_) and the peak of secondary (returned) pressure wave (*P*_2_); the shorter the return time the higher central systolic pressure augmentation
ED: Duration of ejection from the left ventricle during systole
DRA: Diastolic reflection area, estimated area under the pressure wave which shows the contribution of reflected pressure wave to diastolic coronary perfusion pressure
SAI: Systolic area index expresses the systolic proportion of the heart cycle
DAI: Diastolic area index expresses the diastolic proportion of the heart cycle.
